# Topological constraints of structural elements in regulation of catalytic activity in HDV-like self-cleaving ribozymes

**DOI:** 10.1038/srep28179

**Published:** 2016-06-15

**Authors:** Chiu-Ho T. Webb, Dang Nguyen, Marie Myszka, Andrej Lupták

**Affiliations:** 1 Department of Molecular Biology and Biochemistry, University of California, Irvine, CA 92697, USA; 2 Department of Chemistry, University of California, Irvine, CA 92697, USA; 3 Department of Pharmaceutical Sciences, University of California, Irvine, CA 92697, USA

## Abstract

Self-cleaving ribozymes fold into intricate structures, which orient active site groups into catalytically competent conformations. Most ribozyme families have distinct catalytic cores stabilized by tertiary interactions between domains peripheral to those cores. We show that large hepatitis delta virus (HDV)-like ribozymes are activated by peripheral domains that bring two helical segments, P1 and P2, into proximity – a “pinch” that results in rate acceleration by almost three orders of magnitude. Kinetic analysis of ribozymes with systematically altered length and stability of the peripheral domain revealed that about one third of its free energy of formation is used to lower an activation energy barrier, likely related to a rate-limiting conformational change leading to the pre-catalytic state. These findings provide a quantitative view of enzyme regulation by peripheral domains and may shed light on the energetics of allosteric regulation.

Self-cleaving ribozymes, which catalyze site-specific scission of their phosphodiester backbone, are the smallest catalytic RNAs found in nature. They fold into a variety of active structures, including a three-way junction in the hammerhead ribozyme, a four-way junction in the hairpin ribozyme, and a double-pseudoknot in the hepatitis delta virus (HDV) ribozymes^1^. In the hammerhead and hairpin ribozymes, regions distant from the cores form tertiary interactions that lock the helices into restricted conformations and bias the structures into catalytically-competent states, but similar stabilization by peripheral domains has not been proposed for the HDV family of ribozymes.

HDV-like self-cleaving ribozymes are ubiquitous catalytic RNAs, with biological roles ranging from processing of sub-viral transcripts and non-LTR retrotransposons to potential translation initiation of retrotransposon mRNAs^2^,^3^,^4^,^5^. In mammals these ribozymes map to the *CPEB3* gene and have been implicated in episodic memory formation^6^,^7^. Paired regions in these ribozymes fold into two co-axial helical stacks (P1 stacks with P1.1 and P4, P2 stacks on P3) to form a nested double-pseudoknot (Fig. 1a)^8^,^9^,^10^. The cleavage site maps to the first nucleotide of the P1 helix. The catalytic core is formed by single-stranded regions L3 and J4/2, and the nucleotides at the base of the stacked P2-P3 helices. Correct orientation of the P1 and P2-P3 helical segments is therefore crucial for catalysis.

The peripheral ends of the P1 and P2 are connected by the joining strand J1/2 and do not form tertiary contacts to lock the helical segments into parallel orientation. Crystal structures of the precursor and product forms of the genomic HDV ribozyme showed similar overall structures in both *cis*- and *trans*-cleaving constructs, with the active site largely preorganized for RNA scission^8^,^9^,^10^. In contrast, FRET-based solution experiments on a *trans*-cleaving construct, which lacks the J1/2 linker, showed an increase in distance between the distal ends of P2 and P4 upon ribozyme cleavage, thus undergoing a conformational change between the precursor and product states^11^ concomitant with a local conformational change in the ribozyme core, suggesting that the global conformational change translates to local conformation of the active site with matching dynamics^12^,^13^,^14^. Furthermore, *trans*-acting HDV ribozymes cleave their substrates slower than *cis*-acting constructs, even at saturating substrate concentrations^15^,^16^, and a 2-aminopurine placed adjacent to the active site shows different fluorescence lifetimes for the *cis* and trans constructs^14^. Tb^3+^ footprinting studies indicated that the J1/2 region becomes more inaccessible during the course of the reaction, straining the J1/2 linker strand^17^, and Fe^2+^-EDTA footprinting and RNase cleavage showed that both the distal termini of P1 and P2, together with the J1/2 linker, become inaccessible upon ribozyme self-scission^18^. Finally, kinetic competition between ribozyme conformational changes and antisense oligonucleotide binding during the course of self-scission showed progressive closing of the P1-J1/2-P2 region^19^. These observations support a relaxed precursor state of the ribozyme, in which the distal termini of the P2 and P4 helices are closer and implying that the P1 and P4 helices are not parallel (juxtaposed). Because the cleavage rate constant of the *trans* ribozyme is lower than the equivalent self-cleaving form, this proposed relaxed precursor state must affect the formation of the catalytically-competent active site. Indeed, ribozymes lacking the crossover J1/2 strand appear to have larger hydrodynamic radii and are more dynamic than equivalent constructs containing intact J1/2 strands^20^, further supporting a model of a relaxed pre-catalytic state.

The discovery of large, naturally-occurring HDV-like ribozymes containing additional domains in the J1/2 region suggested that this segment can be readily altered while maintaining cleavage activity. Ribozymes with J1/2 domains predicted to form stable structures exhibited fast self-scission^4^,^21^. Based on these observations, we hypothesized that fast, *cis*-cleaving HDV-like ribozymes promote catalysis by bringing the P1 and P2 helices into proximity either by a short J1/2 linker or a stable domain, assisting in formation of the catalytic core. To test this hypothesis, we studied the mosquito ribozyme drz-Agam-2-1 (Fig. 1a), an HDV-like ribozyme that self-cleaves in seconds at physiological conditions^21^.

## Results

**Figure 1:**
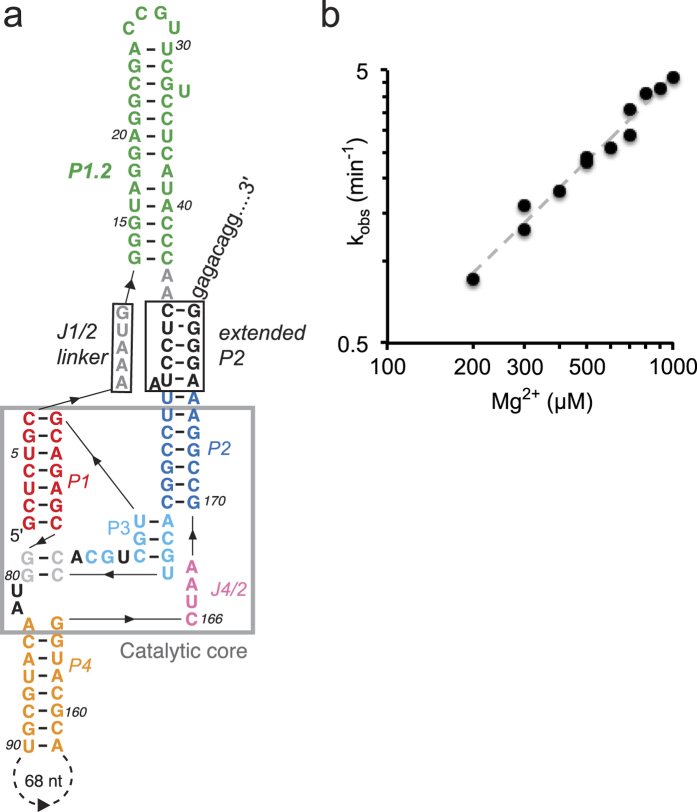
Secondary structure and activity of an HDV-like ribozyme with a structured J1/2-P1.2 domain. (**a**) *A. gambiae* Agam-2-1 HDV-like ribozyme. The catalytic core of the ribozyme is boxed in grey, the RNA self-cleaves at its 5′ terminus. (**b**) Log-log plot of Mg^2+^-dependence of the ribozyme reveals a Hill coefficient of 1.06 (see Materials and Methods).

We began by measuring the Mg^2+^ dependence of the Agam-2-1 ribozyme. We found that this sequence requires Mg^2+^ for self-scission, but exhibits no cooperativity (Fig. 1b). This result is in stark contrast to other ribozymes of this family, which typically show a Hill coefficient of 1.7–2 in activity-*versus*-Mg^2+^ plots^6^,^22^,^23^,^24^. In HDV ribozymes, one divalent metal ion binds in the active site^9^,^10^ and is thought to be directly involved in catalysis, possibly through several mechanisms that include coordination of a hydroxide that deprotonates the 2′ nucleophile, direct stabilization of the 2′ nucleophile, and binding the phosphorane transition state^25^,^26^. The second Mg^2+^, which gives rise to the Hill coefficient larger than unity, is likely to be an anion stabilizing the catalytically-competent structure of these ribozymes^27^,^28^. With a Hill coefficient of 1.06, the Agam-2-1 ribozyme appears to require only the catalytic Mg^2+^, suggesting that a stable J1/2 domain alleviates the strong dependence of the ribozyme on the second divalent metal ion.

**Figure 2:**
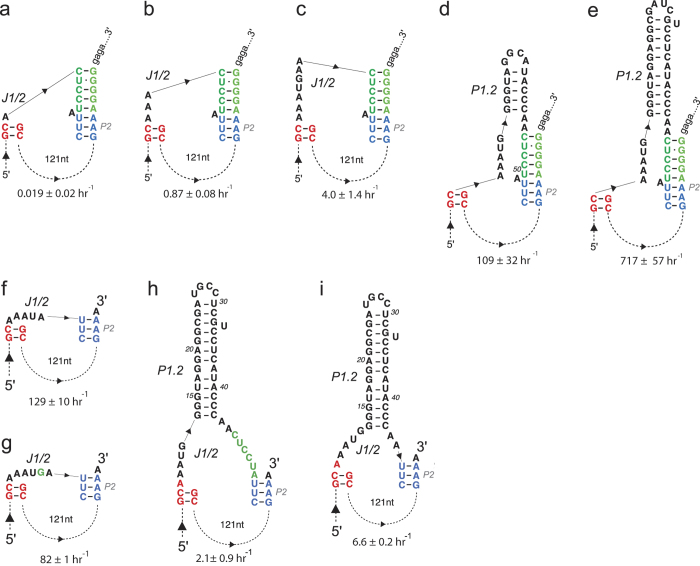
Effect of J1/2 domain on the activity of the Agam-2-1 ribozyme. At physiological-like conditions incremental lengthening of the J1/2 strand results in progressively faster self-scission (**a–c**) and restoring the P1.2 helix increases cleavage rates further (**d,e**). (**f**) Removing the extended P2 as well as the P1.2 helices results in slower self-scission and lengthening the J1/2 linker slows it further (**g**). (**h**) Reintroducing the full-length P1.2 helix connected by long linkers further slows down the ribozyme activity, which is partially restored by shortening the linker on the P2 side (**i**). All constructs, including intermediate ones not shown here, are listed in the Supplementary Table S1. No core parts of the ribozymes were altered in these constructs.

To investigate the influence of this large element on the ribozyme activity, we prepared deletion constructs in the J1/2 domain and measured their effect on self-scission. The secondary structures were calculated using the thermodynamic parameters for pseudoknots^29^,^30^, correctly predicting the overall pseudoknot of *CPEB3* (recently confirmed by NMR methods)^31^ and HDV ribozymes, but not the one or two base-pairs of the P1.1, which defines the second pseudoknot (Supplementary Table S1). There was no activity in the absence of a spacer between P1 and P2 and little self-scission was recovered with a single nucleotide spacer (Fig. 2a; see Supplementary Table S1 for all constructs and activities), apparently restricting the formation of competent catalytic cores. With longer (3- and 7-nt) spacers, the cleavage rate constants gradually increased (Fig. 2b,c) to several per hour. A dramatic rate jump of almost 100-fold was observed when a base-paired P1.2 structure was reintroduced (Fig. 2d), while keeping the single-stranded linker length the same. The rate increased further with the length of the P1.2 helix (Fig. 2e; and Supplementary Table S1). Control constructs, predicted to form long P1.2-like domains that interfere with the formation of the P2 helix, or are predicted to cause alternative base-pairing in other parts of the ribozyme, showed no detectable activity. Our data thus show that at minimum a short spacer is needed between P1 and P2, and a stable base-paired J1/2 region structure strongly promotes the cleavage reaction.

**Figure 3:**
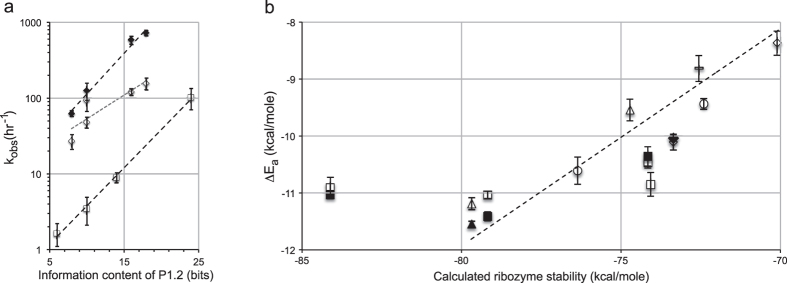
Analysis of the influence of the P1.2 domain on the ribozyme activity. **(a)** Activity-information relationship in Agam-2-1 ribozyme variants with variable number of base-pairs in P1.2 helix. Self-cleavage rate constants from 1 mM (solid) and 0.3 mM (open) Mg^2+^ experiments are plotted as a function of information content of the P1.2 helix. The information content of a base-pair is taken to be 2 bits. The 1 mM dataset exhibits a linear trend with ~12-fold increase in rate constants for each additional 10 bits of information content in P1.2 (5 additional base-pairs). The 0.3 mM Mg^2+^ data fall into two groups showing ~10-fold (squares) and 5.2-fold (diamonds) increase in k_obs_ per 10 additional bits of information content. (**b**) Activity dependence on the ribozyme stability, plotted as a change in activation energy (ΔE_a_) *vs.* mean free energy of the structure of each ribozyme construct when both the length and composition of P1.2 helix are altered. Each symbol corresponds to a unique pair of connecting linkers at either 1 mM Mg^2+^ (solid) or 300 μM Mg^2+^ (open) (see Supplementary Table 1 for all constructs and predicted secondary structures). Error bars represent average deviations for at least two experiments. Dashed line represents a linear fit to all data other than for the wild-type (WT) sequence (slope = 0.38).

The catalytic enhancement by a stable J1/2 structure appears to arise largely from juxtaposition of the core helices and suggests that the strands linking the J1/2 domain to P1 and P2 may have a similar effect. Most HDV-like ribozymes contain a short J1/2 strand connecting a 6- or 7-bp P1 helix with an ~7-bp P2 helix, which extends to 12 bps in Agam-2 ribozymes^21^,^23^. To analyze the influence of the extended P2 helix, we shortened it from the 12 bps seen in the wild-type Agam-2 ribozymes, to 7 bps. In a construct with a short 5-nt J1/2 linker, similar in structure and kinetics to the HDV and the chimp *CPEB3* ribozymes^32^, we observed fast self-scission of >1 min^−1^ (Fig. 3f). The activity decreased when the linker was extended to 6 nt (Fig. 3g) and introducing a full-length, structured P1.2 into this construct did not rescue rapid self-scission in the absence of the extended P2 (Fig. 3h). The resulting construct, with 12-nts of linker between P1 and the shorter P2, led to very slow catalysis, requiring about half an hour to self-cleave – a decrease by ~175 times when compared to the wild-type construct with both a P1.2 helix and a fully extended P2 (Fig. 1a). A hybrid construct, consisting of the shortened 7-bp P2 and full-length P1.2, but connected by shorter 5- and 2-nt J1/2 linker strands, restored self-cleavage rate by about 3 times (Fig. 3i). Together these data demonstrate that a structured P1.2 is not sufficient for activation of the ribozyme. A combination of short single-stranded linker, P1.2 structure, and extended P2 helix results in “pinching” of the P1 and P2 elements together into the catalytically active conformation.

To measure the contribution that the P1.2 helical domain makes toward activation of the ribozyme, we designed a series of constructs that differed only in the predicted stability of the J1/2 domain and measured their activity in 0.3 and 1 mM Mg^2+^. As noted above, the more stable J1/2 domains supported faster self-scission. An analysis based on the number of base-pairs in P1.2 revealed a linear trend (Fig. 3a), but only for constructs with similar predicted J1/2-P1.2 structure, i.e. similar linkers but variable P1.2 length. Interestingly, for these related constructs the plot reveals an ~10-fold increase in activity for a 5-bp lengthening of P1.2, corresponding to 10 additional bits of information, and matching the information–activity relationship previously established for *in vitro* selected RNAs using molecular phylogeny^33^, rather than thermodynamic stability used here.

Because the progressive stabilization of the P1.2 helix leads to faster observed rate constants, we hypothesized that the domain affects a rate-limiting conformational change that leads to the activated, pre-catalytic ribozyme. To extract the thermodynamic contribution of the peripheral domain to lowering the activation energy of the rate-limiting step, we plotted the apparent activation energy (calculated as –RT^*^ln[k_obs_/k_uncat_]) against the predicted stability of the entire ribozyme. Here k_obs_ is the measured self-scission rate constant, which likely reflects the kinetics of a conformational change as well as the transphosphorylation reaction, and k_uncat_ is the background rate of RNA degradation at the appropriate Mg^2+^, K^+^, pH, and temperature, calculated based on rate constants established by Li and Breaker^34^. As noted above, the predicted secondary structures and thermodynamic stabilities were calculated using the thermodynamic parameters for pseudoknots^29^,^30^. The graph (Fig. 3b) revealed a linear relationship between the activation energy and stability of P1.2 helices. The data fit to a line with a slope of 0.38, suggesting that about one third of the free energy of P1.2 formation is used to juxtapose P1 and P2 and promote formation of the activated pre-catalytic state of the ribozyme. Once the P1.2 helix contributes more than ~4 kcal/mol (~17 kJ/mol), the plot levels off, indicating that further increase in the stability of the peripheral domain does not contribute to activation of the catalytic core, or that the activation step is no longer rate-limiting. The total energy contribution by the peripheral domain results in almost three orders of magnitude of reaction acceleration at 37 °C.

The fastest P1.2-containing Agam-2 ribozyme (Fig. 2e) self-cleaves with k_obs_~0.2 sec^−1^ in 1 mM Mg^2+^, which is likely ~2 sec^−1^ in 10 mM Mg^2+^, given the Hill coefficient of unity and absence of saturation observed in Fig. 1b. The fastest constructs thus match the self-cleavage rates of HDV ribozymes engineered to have optimal folding with the rate-limiting step being chemistry, and not a conformational transition^35^, whereas the kinetics of the slower constructs, with weaker P1.2 helices or longer spacers in J1/2, appear to be dominated by a conformational change. The 4 kcal/mol contribution that P1.2 makes to acceleration of the observed rate of self-scission thus appears to lower the activation barrier of the conformational change leading to the pre-catalytic, activated ribozyme to a point where the barrier is no longer rate-limiting and revealing the activation barrier related to catalysis of phosphoryl transfer.

## Discussion

Our experiments demonstrate that a structured domain between P1 and P2 helices in HDV-like ribozymes promotes catalysis. Before our discovery of a long J1/2 insert region in the mosquito ribozymes, a structured J1/2 was shown to stabilize the P2 helix in an engineered *trans*-ribozyme with an additional 7 base-pair stem (P1.2-like) in the J1/2 region^36^. The J1/2 region was also used to design a specific on/off adaptor system for a *trans* HDV ribozyme, which acts as a switch with the ribozyme “on” only when an allosteric substrate is present^37^. Moreover, using footprinting and FRET assays, the J1/2 region was implicated as the critical element that determines the different structural characteristics between the *cis*- and *trans*-acting HDV ribozymes^20^. In contrast, crystal structures of *cis*- and *trans*-acting HDV ribozymes show similar overall structure^8^,^9^,^10^, suggesting that the ribozymes undergo limited global conformational change during catalysis. However, in the *trans* HDV ribozyme structure lacking a J1/2 strand, the P1 helix of one molecule forms a crystal contact with another molecule and this section of the P1 helix has the most interpretable electron density^9^, suggesting higher disorder in the rest of the substrate helix (including the cleavage site). It is therefore likely that the *trans* ribozyme crystallized in an activated conformation stabilized by crystal packing forces and not in the relaxed pre-catalytic state.

**Figure 4:**
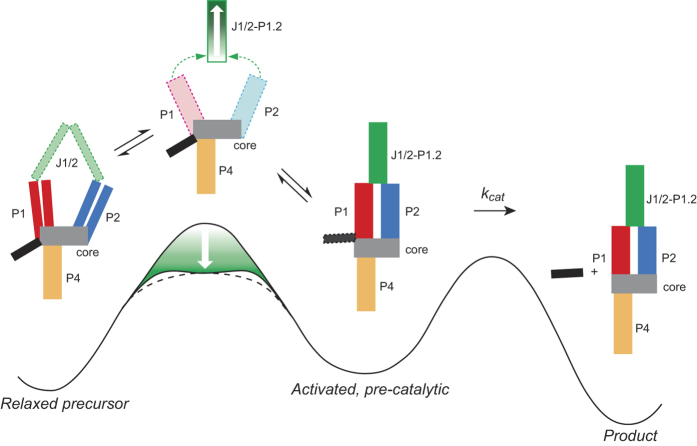
A “pinch” model for activation of HDV-like ribozymes with variable J1/2-P1.2 domain. The relaxed precursor state with P1 and P2 apart and unfolded P1.2 is shown on the left above the energy graph for the proposed reaction coordinate. The activation barrier for the conformational change that leads to the activated, pre-catalytic state, destabilized by ~2 kcal/mol^38^, depends on the strength of the P1.2 helix or the length of the J1/2 linker. The strength of the P1.2 helix (green gradient with white arrow depicting progressively more stable P1.2) correlates with lowering of the activation barrier (white arrow on the green gradient of the left energy barrier) by up to ~4 kcal/mol to either form an intermediate (black line, bottom of the gradient) or a lower transition state (dashed line), which corresponds to the low conformational transition state in optimally folding HDV ribozymes^35^. Stabilization of P1.2 causes “pinching” of P1 and P2 into juxtaposition (green dashed arrows), leading to the formation of the activated ribozyme, which self-cleaves (k_cat_) at the 5′ end of P1 (black-red interface) and quickly dissociates from the 5′ sequence (black). Domains proposed to undergo conformational change are depicted with dashed outlines. The energy levels are not drawn to scale.

To explain the kinetic behavior of P1.2-containing ribozymes, we propose a model for the self-scission reaction, in which the ribozymes are initially in an inactive state with the distal termini of P1 and P2 apart, and possibly partially unpaired, as suggested by biochemical data on constructs lacking the J1/2 strands^11^,^12^,^13^,^14^,^17^,^18^,^19^ and the observation that in the *trans* construct of the antigenomic HDV ribozyme the dissociation rate of P1 is much faster in the precursor than in product forms^15^. Our model builds on previously proposed models for activation of HDV ribozymes^35^,^38^, incorporating a rate-limiting conformational barrier (Fig. 4, green gradient). Upon addition of mono- and divalent metal ions, the ribozymes undergo a conformational change, juxtaposing P1 and P2 to form a catalytically-competent intermediate, activated by about 2 kcal/mol relative to the relaxed state^38^, and leading to the chemical step (Fig. 4). Ribozymes with short J1/2 linkers or stable P1.2 domains promote formation of this intermediate by lowering the activation energy of the conformational change to a point where the transition is no longer rate-limiting (Fig. 4; longer P1.2 and corresponding lower transition state energies are depicted by white arrows in the structural model and energy landscape, respectively) and k_obs_ ≈ k_chem_ ≈ 1 sec^−1^ or faster^35^, and putting mechanical strain on the J1/2 regions, as proposed previously^17^. Ribozymes with longer J1/2 strands or weaker P1.2 domains bring P1 and P2 into proximity significantly slower, suggesting that they lower the activation energy barrier for the conformational change less than the highly active ribozymes and this conformational change is rate-limiting. This point also suggests a hierarchy of folding, during which the P1.2 forms first to bring together (or “pinch”) P1 and P2 helices, which in turn drive the formation of a catalytically competent active site, as suggested by FRET and active-site fluorescence kinetics^12^,^14^. Moreover, if P1.2 forms before other parts of the ribozyme fold into the activated structure, the lowering of the conformational barrier may coincide with the formation of a partially stable intermediate, as shown in Fig. 4. At this point, we have no experimental evidence suggesting the formation of this intermediate, which we hope to investigate by time-resolved structural probing methods in the future.

Because the pre-catalytic intermediate state of the ribozyme appears to be strained^17^, we expect the P1.2 domain to primarily affect the transition state energy of the conformational change, with less of an effect on the stability of the intermediate. In other words, we do not expect the relative energies of the precursor, activated, and product states to be significantly altered by the P1.2 domain and the equilibrium between the relaxed and pre-catalytic state are likely to be unaffected by the P1.2 structure. Our kinetic data for the P1.2 series of constructs do not show any significant trend in uncleaved fraction of the ribozymes or relative populations of fast and slow cleaving sub-populations (data not shown), supporting the hypothesis that the relative energies of the precursor, activated intermediate, and product states are similar for ribozymes that differ only in the length of P1.2. The chemistry step in HDV-like ribozymes is unidirectional, because the 5′ (leader) sequence dissociates and renders the reaction practically irreversible^15^. We therefore could not measure the effect of the P1.2 domain on the equilibrium of the overall reaction.

Our data support a model of activation of HDV-like ribozymes by a peripheral domain that lowers the activation energy barrier for a rate-limiting conformational change. The activation energy appears to be decreased by the P1.2 domain such that ~0.38 of the domain’s predicted stability can be utilized for this mechanism. The effect on the rate constant of the rate-limiting conformation change is


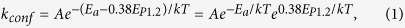


where A is the Arrhenius pre-exponential factor, E_a_ is the activation energy of a construct with long, unstructured peripheral domain, and E_P1.2_ is the predicted energy of formation of the P1.2 domain. The observed rate constant for the ribozyme self-scission when the conformational change is rate-limiting is thus increased by a factor of 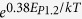.

Taken together, our results on the influence of J1/2 structure show that the formation of a productive catalytic core in the ribozyme can strongly depend on regions remote from the site of catalysis. The large sensitivity and log-linear dependence of the ribozyme activity on the stability of the J1/2 region makes this domain an ideal site for engineering allosteric effector domains^39^,^40^,^41^. To explain the effect of a structured J1/2 domain on catalysis, we propose a model, in which proper positioning of the P1 and P2 helices directs the substrate phosphodiester and the active site into an activated conformation. A helix in the J1/2 linker acts to “pinch” the peripheral termini of P1 and P2 into proximity using about a third of its stabilization energy. When applied to the case of a regulatory-ligand binding, the linear relationship between the peripheral domain’s stability and activation energy change may represent a model for the energetics of allosteric regulation.

## Materials and Methods

### DNA Template Preparation

Individual DNA templates less than 100 bp long were ordered from Integrated DNA Technologies (IDT). Larger products were prepared using primer extension, starting from pieces ordered from IDT. All oligonucletides were PAGE-purified and stored at −20 °C prior to use. Polymerase chain reaction (PCR) reactions were carried out using commercial 2xDreamTaq MasterMix (Invitrogen) preparations in 50 μL reactions. The primer-extension conditions were 94 °C for 1 min, followed by 2 cycles of 50 °C for 30 sec and 72 °C for 2 min. Amplification steps used a PCR program of 94 °C for 1 min, followed by 20 cycles of 50 °C for 30 sec and 72 °C for 2 min. The resulting templates contained a T7 promoter for use in transcription.

### RNA transcription and radiolabeling

RNA was transcribed typically in 5–10 μl containing 10 mM of DTT, 2.5 mM each GTP, UTP and CTP, 250 μM ATP, 1.25 μCi [α-^32^P]-ATP (Perkin Elmer, Waltham, MA), 7.75 mM MgCl2, 20 μM of inhibitor oligo (specific for each type of construct), 1 unit of T7 RNA polymerase, and 0.5 pmole of DNA template. These mixtures were incubated at 37 °C for an hour before quenching with excess EDTA and fractionated by denaturing polyacrylamide gel electrophoresis (PAGE). The resulting bands were excised and shaken in water to elute, then further purified by size exclusion chromatography (G25 Sephadex) twice to remove remaining EDTA before storage at −80 °C.

### Cleavage Kinetics

*In vitro* self-cleavage reactions were performed as follows. Separate solutions of the PAGE-purified ^32^P-labeled ribozyme (6 μL) and a physiological-like MgCl_2_ buffer (25 μL of 140 mM KCl, 10 mM NaCl, 50 mM Tris-HCl, pH 7.5) were pre-incubated at 37 °C. A zero timepoint was collected by taking 1 μL of the ribozyme solution and immediately quenching prior to addition of any magnesium by adding it to 6 μL of denaturing quench buffer (8 M urea, 20 mM EDTA, with 0.05% xylene cyanol, 0.1% bromophenol blue, and 1 mM Tris, pH 7.5). Folding and self-cleaving reactions were initiated by adding the ribozyme (5 μL) to the reaction buffer (25 μL). Aliquots (3 μL) taken at different time points were quenched with 6 μL of the denaturing quench buffer. The reactions were then fractionated using 7.5% PAGE. The resulting gels were exposed to phosphorimage screens (Molecular Dynamics) and analyzed by using a Typhoon phosphorimager. ImageQuant software (GE Healthcare) was used to measure observed band intensities to derive the population of each fragment size at a given timepoint. These data were used to model the progress of the initiated cleavage reaction over time.

### Modeling reaction kinetics

The numerical fraction intact was used to fit equations corresponding to both monoexponential (sinlge rate constant) and biexponential decay (see equation below) to determine which fit the observed data more closely. The observed rate constant for self-cleavage was taken to be the faster one in the case of biexpontential fits.


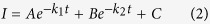


### Determining expected effect on activation energy

The effects of changing the J1/2 and linker regions in the ribozymes under study were determined through the comparison of the observed reaction rate constant to the uncatalyzed rate (k_uncat_) of cleavage for a general phosphodiester bond, as established by Li & Breaker^34^ at 37 °C, 150 mM K^+^ (to account for both K^+^ and Na^+^ in the kinetics experiment), pH 7.5, and either 0.3 or 1 mM Mg^2+^.

The activation energy, E_a_, is defined as


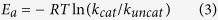


### Minimum free energy (MFE) calculations

The MFE for each form of ribozyme studied was determined by computational calculation using the DotKnot software (available at http://dotknot.csse.uwa.edu.au/) using predictions for local psuedoknots and kissing hairpins. The full sequence of the transcribed ribozyme was input and used as the basis for the contribution that each J1/2-P1.2 construct contributes to the ribozyme structure. Using just the calculated energy of the J1/2-P1.2 domain or the whole ribozyme, including the leader sequence and 3′ tail, gave similar relationship to the one shown in Fig. 2g. The algorithm predicts the overall pseudoknot structure correctly, but does not predict the formation of P1.1, which defines the second pseudoknot, and predicts slightly shifted secondary structure in P3 and 1-nt longer P1 helix. We only considered ribozyme constructs that showed the same predicted core secondary structure as the wild-type ribozyme.

### Hill equation

The cooperativity of Mg^2+^ binding shown in Fig. 1b was taken to be a measure of Mg^2+^ dependence for the ribozyme, modeled as a Hill coefficient (n). This was initially obtained by fitting obtained data to


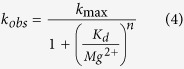


however, since saturation was not observed and k_max_ could not be reliably estimated, we fit the plot of log (k_obs_) *vs* log [Mg^2+^] to a line with the slope of n. The calculated slope in Fig. 1b was 1.02. This approximation of the Hill coefficient only holds if the range of Mg^2+^ concentrations is significantly lower than the K_d_ (K_1/2, Mg_). Similarly, assuming a K_d_ is well above the range, the data can be analyzed directly using a non-linear fit of the k_obs_ equation presented above. This non-linear fit reveals a Hill coefficient of 1.06, reported in the Results section.

## Additional Information

**How to cite this article**: Webb, C.-H. T. *et al*. Topological constraints of structural elements in regulation of catalytic activity in HDV-like self-cleaving ribozymes. *Sci. Rep.* **6**, 28179; doi: 10.1038/srep28179 (2016).

## Supplementary Material

Supplementary Information

## References

[b1] Jimenez R. M., Polanco J. A. & Luptak A. Chemistry and Biology of Self-Cleaving Ribozymes. Trends Biochem Sci 40, 648–661, 10.1016/j.tibs.2015.09.001 (2015). 26481500 PMC4630146

[b2] Been M. D. HDV ribozymes. Curr Top Microbiol Immunol 307, 47–65 (2006). 16903220 10.1007/3-540-29802-9_3

[b3] Eickbush D. G. & Eickbush T. H. R2 retrotransposons encode a self-cleaving ribozyme for processing from an rRNA cotranscript. Mol Cell Biol 30, 3142–3150, 10.1128/MCB.00300-10 (2010). 20421411 PMC2897577

[b4] Ruminski D. J., Webb C. H., Riccitelli N. J. & Luptak A. Processing and translation initiation of non-long terminal repeat retrotransposons by hepatitis delta virus (HDV)-like self-cleaving ribozymes. J Biol Chem 286, 41286–41295, 10.1074/jbc.M111.297283 (2011). 21994949 PMC3308841

[b5] Sanchez-Luque F. J., Lopez M. C., Macias F., Alonso C. & Thomas M. C. Identification of an hepatitis delta virus-like ribozyme at the mRNA 5′-end of the L1Tc retrotransposon from Trypanosoma cruzi. Nucleic Acids Res 39, 8065–8077, 10.1093/nar/gkr478 (2011). 21724615 PMC3185411

[b6] Salehi-Ashtiani K., Luptak A., Litovchick A. & Szostak J. W. A genomewide search for ribozymes reveals an HDV-like sequence in the human CPEB3 gene. Science 313, 1788–1792, 10.1126/science.1129308 (2006). 16990549

[b7] Vogler C. . CPEB3 is Associated with Human Episodic Memory. Front Behav Neurosci 3, 4, 10.3389/neuro.08.004.2009 (2009). 19503753 PMC2691156

[b8] Ferre-D’Amare A. R., Zhou K. & Doudna J. A. Crystal structure of a hepatitis delta virus ribozyme. Nature 395, 567–574, 10.1038/26912 (1998). 9783582

[b9] Chen J. H. . A 1.9 A crystal structure of the HDV ribozyme precleavage suggests both Lewis acid and general acid mechanisms contribute to phosphodiester cleavage. Biochemistry 49, 6508–6518, 10.1021/bi100670p (2010). 20677830

[b10] Ke A., Zhou K., Ding F., Cate J. H. & Doudna J. A. A conformational switch controls hepatitis delta virus ribozyme catalysis. Nature 429, 201–205, 10.1038/nature02522 (2004). 15141216

[b11] Pereira M. J., Harris D. A., Rueda D. & Walter N. G. Reaction pathway of the trans-acting hepatitis delta virus ribozyme: a conformational change accompanies catalysis. Biochemistry 41, 730–740 (2002). 11790094 10.1021/bi011963t

[b12] Harris D. A., Rueda D. & Walter N. G. Local conformational changes in the catalytic core of the trans-acting hepatitis delta virus ribozyme accompany catalysis. Biochemistry 41, 12051–12061 (2002). 12356305 10.1021/bi026101m

[b13] Tanaka Y. . Cleavage reaction of HDV ribozymes in the presence of Mg2+ is accompanied by a conformational change. Genes to Cells 7, 567–579, 10.1046/j.1365-2443.2002.00541.x (2002). 12059960

[b14] Gondert M. E., Tinsley R. A., Rueda D. & Walter N. G. Catalytic core structure of the trans-acting HDV ribozyme is subtly influenced by sequence variation outside the core. Biochemistry 45, 7563–7573, 10.1021/bi052116j (2006). 16768452

[b15] Shih I. & Been M. D. Kinetic scheme for intermolecular RNA cleavage by a ribozyme derived from hepatitis delta virus RNA. Biochemistry 39, 9055–9066 (2000). 10924098 10.1021/bi000499+

[b16] Ananvoranich S. & Perreault J. P. Substrate specificity of delta ribozyme cleavage. J Biol Chem 273, 13182–13188 (1998). 9582360 10.1074/jbc.273.21.13182 PMC2922200

[b17] Harris D. A., Tinsley R. A. & Walter N. G. Terbium-mediated footprinting probes a catalytic conformational switch in the antigenomic hepatitis delta virus ribozyme. J Mol Biol 341, 389–403, 10.1016/j.jmb.2004.05.074 (2004). 15276831

[b18] Savochkina L., Alekseenkova V., Belyanko T., Dobrynina N. & Beabealashvilli R. RNase footprinting demonstrates antigenomic hepatitis delta virus ribozyme structural rearrangement as a result of self-cleavage reaction. BMC Res Notes 1, 15, 10.1186/1756-0500-1-15 (2008). 18710542 PMC2518280

[b19] Wrzesinski J., Legiewicz M. & Ciesiolka J. Mapping of accessible sites for oligonucleotide hybridization on hepatitis delta virus ribozymes. Nucleic Acids Res 28, 1785–1793 (2000). 10734198 10.1093/nar/28.8.1785 PMC102829

[b20] Tinsley R. A. & Walter N. G. Long-range impact of peripheral joining elements on structure and function of the hepatitis delta virus ribozyme. Biol Chem 388, 705–715, 10.1515/BC.2007.088 (2007). 17570823

[b21] Webb C. H., Riccitelli N. J., Ruminski D. J. & Luptak A. Widespread occurrence of self-cleaving ribozymes. Science 326, 953, 10.1126/science.1178084 (2009). 19965505 PMC3159031

[b22] Cerrone-Szakal A. L., Chadalavada D. M., Golden B. L. & Bevilacqua P. C. Mechanistic characterization of the HDV genomic ribozyme: the cleavage site base pair plays a structural role in facilitating catalysis. RNA 14, 1746–1760, 10.1261/rna.1140308 (2008). 18658121 PMC2525964

[b23] Riccitelli N. Lupták D. E., A. Identification of minimal HDV-like ribozymes with unique divalent metal ion dependence in the human microbiome. Biochemistry 53, 1616–1626, 10.1021/bi401717w (2014). 24555915

[b24] Wrzesinski J. L. M., Smólska B. & Ciesiolka J. Catalytic cleavage of cis- and trans-acting antigenomic delta ribozymes in the presence of various divalent metal ions. Nucleic Acids Res 29, 4482–4492 (2001). 11691936 10.1093/nar/29.21.4482 PMC60188

[b25] Das S. R. & Piccirilli J. A. General acid catalysis by the hepatitis delta virus ribozyme. Nat Chem Biol 1, 45–52, 10.1038/nchembio703 (2005). 16407993

[b26] Thaplyal P., Ganguly A., Golden B. L., Hammes-Schiffer S. & Bevilacqua P. C. Thio effects and an unconventional metal ion rescue in the genomic hepatitis delta virus ribozyme. Biochemistry 52, 6499–6514, 10.1021/bi4000673 (2013). 24001219 PMC3825741

[b27] Nakano S., Cerrone A. L. & Bevilacqua P. C. Mechanistic characterization of the HDV genomic ribozyme: classifying the catalytic and structural metal ion sites within a multichannel reaction mechanism. Biochemistry 42, 2982–2994, 10.1021/bi026815x (2003). 12627964

[b28] Skilandat M., Rowinska-Zyrek M. & Sigel R. K. Solution structure and metal ion binding sites of the human CPEB3 ribozyme’s P4 domain. J Biol Inorg Chem 19, 903–912, 10.1007/s00775-014-1125-6 (2014). 24652468

[b29] Kopeikin Z. & Chen S. J. Folding thermodynamics of pseudoknotted chain conformations. J Chem Phys 124, 154903, 10.1063/1.2188940 (2006). 16674261 PMC2442620

[b30] Sperschneider J. & Datta A. DotKnot: pseudoknot prediction using the probability dot plot under a refined energy model. Nucleic Acids Res 38, e103, 10.1093/nar/gkq021 (2010). 20123730 PMC2853144

[b31] Skilandat M., Rowinska-Zyrek M. & Sigel R. K. Secondary structure confirmation and localization of Mg2+ ions in the mammalian CPEB3 ribozyme. RNA, 10.1261/rna.053843.115 (2016). PMC4836649 26966151

[b32] Chadalavada D. M., Gratton E. A. & Bevilacqua P. C. The human HDV-like CPEB3 ribozyme is intrinsically fast-reacting. Biochemistry 49, 5321–5330, 10.1021/bi100434c (2010). 20524672 PMC2890282

[b33] Carothers J. M., Oestreich S. C., Davis J. H. & Szostak J. W. Informational complexity and functional activity of RNA structures. J Am Chem Soc 126, 5130–5137, 10.1021/ja031504a (2004). 15099096 PMC5042360

[b34] Li Y. F. & Breaker R. R. Kinetics of RNA degradation by specific base catalysis of transesterification involving the 2′-hydroxyl group. J Am Chem Soc 121, 5364–5372, 10.1021/Ja990592p (1999).

[b35] Brown T. S., Chadalavada D. M. & Bevilacqua P. C. Design of a highly reactive HDV ribozyme sequence uncovers facilitation of RNA folding by alternative pairings and physiological ionic strength. J Mol Biol 341, 695–712, 10.1016/j.jmb.2004.05.071 (2004). 15288780

[b36] Hori T., Guo F. & Uesugi S. Addition of an extra substrate binding site and partial destabilization of stem structures in HDV ribozyme give rise to high sequence-specificity for its target RNA. Nucleosides Nucleotides Nucleic Acids 25, 489–501, 10.1080/15257770600684183 (2006). 16838841

[b37] Levesque M. V., Rouleau S. G. & Perreault J. P. Selection of the most potent specific on/off adaptor-hepatitis delta virus ribozymes for use in gene targeting. Nucleic Acid Ther 21, 241–252, 10.1089/nat.2011.0301 (2011). 21793786 PMC5021529

[b38] Shih I. & Been M. D. Energetic contribution of non-essential 5′ sequence to catalysis in a hepatitis delta virus ribozyme. Embo J 20, 4884–4891 (2001). 11532952 10.1093/emboj/20.17.4884 PMC125606

[b39] Bergeron L. J. & Perreault J. P. Target-dependent on/off switch increases ribozyme fidelity. Nucleic Acids Res 33, 1240–1248, 10.1093/nar/gki278 (2005). 15731344 PMC549572

[b40] Kobori S., Nomura Y., Miu A. & Yokobayashi Y. High-throughput assay and engineering of self-cleaving ribozymes by sequencing. Nucleic Acids Res 43, e85, 10.1093/nar/gkv265 (2015). 25829176 PMC4513843

[b41] Bergeron L. J., Reymond C. & Perreault J. P. Functional characterization of the SOFA delta ribozyme. RNA 11, 1858–1868, 10.1261/rna.2112705 (2005). 16251383 PMC1370874

